# Impact of Noncommunicable Disease Multimorbidity on Healthcare Utilisation and Out-Of-Pocket Expenditures in Middle-Income Countries: Cross Sectional Analysis

**DOI:** 10.1371/journal.pone.0127199

**Published:** 2015-07-08

**Authors:** John Tayu Lee, Fozia Hamid, Sanghamitra Pati, Rifat Atun, Christopher Millett

**Affiliations:** 1 Department of Primary Care and Public Health, School of Public Health, Imperial College London, London, United Kingdom; 2 Indian Institute of Public Health, Public Health Foundation of India, Bhubaneswar, India; 3 Department of Global Health and Population, Harvard School of Public Health, Harvard University, Boston, Massachusetts, United States of America; 4 Public Health Foundation of India, Gurgaon, India; Queensland University of Technology, AUSTRALIA

## Abstract

**Background:**

The burden of non-communicable disease (NCDs) has grown rapidly in low- and middle-income countries (LMICs), where populations are ageing, with rising prevalence of multimorbidity (more than two co-existing chronic conditions) that will significantly increase pressure on already stretched health systems. We assess the impact of NCD multimorbidity on healthcare utilisation and out-of-pocket expenditures in six middle-income countries: China, Ghana, India, Mexico, Russia and South Africa.

**Methods:**

Secondary analyses of cross-sectional data from adult participants (>18 years) in the WHO Study on Global Ageing and Adult Health (SAGE) 2007–2010. We used multiple logistic regression to determine socio-demographic correlates of multimorbidity. Association between the number of NCDs and healthcare utilisation as well as out-of-pocket spending was assessed using logistic, negative binominal and log-linear models.

**Results:**

The prevalence of multimorbidity in the adult population varied from 3∙9% in Ghana to 33∙6% in Russia. Number of visits to doctors in primary and secondary care rose substantially for persons with increasing numbers of co-existing NCDs. Multimorbidity was associated with more outpatient visits in China (coefficient for number of NCD = 0∙56, 95% CI = 0∙46, 0∙66), a higher likelihood of being hospitalised in India (AOR = 1∙59, 95% CI = 1∙45, 1∙75), higher out-of-pocket expenditures for outpatient visits in India and China, and higher expenditures for hospital visits in Russia. Medicines constituted the largest proportion of out-of-pocket expenditures in persons with multimorbidity (88∙3% for outpatient, 55∙9% for inpatient visit in China) in most countries.

**Conclusion:**

Multimorbidity is associated with higher levels of healthcare utilisation and greater financial burden for individuals in middle-income countries. Our study supports the WHO call for universal health insurance and health service coverage in LMICs, particularly for vulnerable groups such as the elderly with multimorbidity.

## Introduction

Non-communicable diseases (NCDs) are the leading cause of global disease burden[1, 2], with 80% of NCD mortality occurring in low- and middle-income countries (LMICs)[3, 4]. As the populations in these countries age, the prevalence of multimorbidity, defined as persons with two or more co-existing chronic conditions, will likely increase[5–8]. A recent study in Scotland found that while only 2% of the study population aged less than 25 years had multimorbidity, this percentage increased to nearly 65% for those aged 65 to 84 years[5]. Despite the growing prevalence of NCD multimorbidity in LMICs[9], there is little attention given to the impacts of multimorbidity on individuals and health systems as opposed to single chronic disease[10, 11].

The United Nations High-Level Meeting on NCDs in 2011 stressed the enormous challenge posed by the growing burden of NCDs for health systems in LMICs. Most LMICs have relatively low levels of public expenditure on health and incomplete health insurance and health service coverage[12–15]. The shortfall in public expenditure on health care is typically made up by out-of-pocket (OOP) and other private expenditures on health. According to WHO, private expenditures constituted up to 70% of total health expenditures in India, and more than 40% in Russia and China[16]. The heavily dependence of out-of-pocket expenditure to fund health systems in many LMICs is concerning, as growing evidence suggests user charges adversely affect health, in particular for the elderly and those with chronic illness[17–19]. The impoverishing effect of healthcare related OOP expenditures on individuals and households in LMICs has been well documented[20, 21]. Less is known about the effect of NCD multimorbidity on healthcare utilisation and out-of-pocket expenditures[15, 22].

This study uses nationally representative data from six middle-income countries; China, Ghana, India, Mexico, Russia, and South Africa. Progress toward universal health coverage in these countries has been mixed (see Table A, Fig A in S1 File for country characteristics). For instance, China and Mexico have introduced policies which have resulted in dramatic progress in achieving universal health coverage. After the establishment of the New Cooperative Medical Scheme, the population coverage of health insurance has increased substantially in China, particularly in rural areas. However, patients are still required to pay a large proportion of health expenditures through out-of-pocket payments[23, 24]. At the beginning of the millennium, Mexico had a fragmented health system with several health insurance schemes covering formal workers, government employees and the private sector with a large proportion of the population falling out of the health insurance coverage net[25, 26]. However, since 2003, Mexico implemented a national health insurance scheme (Seguro Popular) which offers health insurance coverage for those not working in formal sector and previously excluded from social health insurance. This scheme has improved coverage of health insurance in the country dramatically. Public spending on health care remains low in India at about 1% of Gross Domestic Product with a minority of the population covered by any form of social or voluntary health insurance[27]. Major national and state level health insurance schemes for the poor, such as Rashtriya Swasthya Bima Yojna (RSBY), do not appear to have addressed the major burden of out of pocket expenditure faced by households in the country[28].

The aim of the study is to look at the socio-demographic correlates of NCD multimorbidity. Furthermore, we examined the impact of NCD multimorbidity on healthcare utilisation and out-of-pocket expenditures. We hypothesise that persons with multimorbidity will have substantially greater need for health care, with higher healthcare utilisation and out-of-pocket expenditures due to their more complex clinical and health needs, than individuals with single NCD[8, 29]. We analyse the source of out-of-pocket expenditures (i.e. medication, healthcare provider fees, medical test) in persons with multimorbidity[30, 31]. We believe this is the first study reporting multimorbidity levels, its socio-economic and demographic correlates, as well as its impact on healthcare utilisation and healthcare expenditures using nationally representative data in multiple middle-income countries.

## Methods

### Sample and data

We used cross-sectional data from the World Health Organization (WHO) Study on Global Ageing and Adult Health (SAGE) collected via nationally representative population surveys in six middle-income countries: China, Ghana, India, Mexico, Russia and South Africa. The survey includes nationally representative cohorts of persons aged 50+ years, with a smaller cohort of persons aged 18 to 49 for comparison purposes[32]. In brief, the aim of the SAGE is to understand self-reported health problems, disability, healthcare utilisation and subjective well-being of adult populations to inform evidence based policy making.[32] The survey objectives and methods are detailed elsewhere.[32]

The data were collected simultaneously in multiple countries during 2007–10, with the total sample size in six countries of 44,464 persons (sample size in China: 14785, Ghana: 5573, India: 12198, Mexico: 2734, Russia: 4947, South Africa: 4227), and allow cross-country comparison of key health indicators.[33, 34] In our analysis, we included respondents aged ≥18 years, excluding those who had missing values on study variables (11∙4% of the sample). As values for out-of-pocket expenditures were highly skewed, we removed observations with the highest 0∙5% of out-of-pocket expenditures to lessen the skewing effects and influence of outliers on the analysis.

### Variables

Our main variable of interest was whether respondents had more than one NCD. In the SAGE, the list of NCDs asked about were: angina, arthritis, asthma, cataracts, diabetes, stroke, chronic lung disease, hypertension and depression. We defined respondents as having an NCD if they answered affirmatively the following two questions: “Have you ever been told by a health professional that you have…? (for example, arthritis)”, or “Have you ever been diagnosed with…?”. We counted the number of NCDs for each respondent, and defined those with multimorbidity as the presence of two or more of the above listed conditions without a specific reference condition.

Respondents were asked about their utilisation of outpatient and inpatient services; whether or not they had any outpatient visit, and number of outpatient visits in the past 12 months; or any overnight hospital stay in the past three years, and number of overnight stays in hospital in the past 12 months. SAGE also collected information on how much respondents paid out-of-pocket for their last outpatient or inpatient visit including the treatment was free of charge. The out-of-pocket expenditures were further categorised by types of service and items, including healthcare provider fees, medicines, medical tests, transport and others. We calculated the proportion of out-of-pocket expenditures on each type of service.

We included the following covariates in the analyses: age (18–29, 30–39, 40–49, 50–59, 60–69, 70+ years), gender, residence (rural, urban), geographical regions (country specific state/region variables), education (no formal education, primary school completed, secondary school and above), health insurance status (with/without insurance), and wealth quintiles.

### Statistical analysis

We used multiple logistic regression analysis to determine socioeconomic and demographic correlates of having any one and multiple NCDs. We calculated the pooled estimate using data from all countries, but also ran separate analyses in each country and reported adjusted odds ratios (AOR) and 95% confidence intervals (CI). We assessed the association between number of NCDs and whether the respondent had any outpatient/inpatient visit (the binary outcome variable) using a logistic regression model, and estimated the number of outpatient visits or hospital stays using negative binomial models.

We summarized the proportion of respondents who reported that their last health care visit was free / incurred no charge. To reduce skewness for data on out-of-pocket expenditures, we used a log-linear model to assess associations between the number of NCDs and spending, where a constant equals to one was added to the outcome variable prior to the log-transformation. We did not undertake a pooled analysis of out-of-pocket expenditure as the variable was measured in each country’s own currency. To examine whether there was differential effect of multimorbidity in different population groups, we conducted subgroup analyses stratified on rural / urban residence and wealth quintile using the same regression model, with stratification variable dropped.

We tested for multicollinearity for covariates adjusted for in our analysis. The multicollinearity diagnostics (Variance Inflation Factor) were all less than five, indicating that the assumption of reasonable independence among predictor variables was met[35]. We presented AOR for results in logistic regression model, and regression coefficient for results in negative binomial and log-linear models. All data analysis was weighted to account for the complex, multi-stage design of the SAGE survey. We performed the statistical analyses using Stata 13∙1 (StataCorp).

## Results

We analysed data from 39,213 respondents from six countries (n = 13,191 in China; 4,873 in Ghana; 11,043 in India; 2,595 in Mexico; 4,268 in Russia; and 3,243 in South Africa). We present respondents’ socioeconomic and demographic characteristics in each country in Table B in S1 File. Overall, in the sample with six countries combined (the pooled analysis), the median age of the respondents was 44∙6 years (IQR = 35–53), 51% were female, 75% were married, and 49% were residing in an urban area. The mean number of NCDs and proportion of persons with multimorbidity was lowest in Ghana (mean 0∙23, with 3∙9% having multimorbidity) and highest in Russia (mean number of NCDs = 1∙22, with 33∙6% had multimorbidity).

### Multimorbidity levels

**Fig 1:**
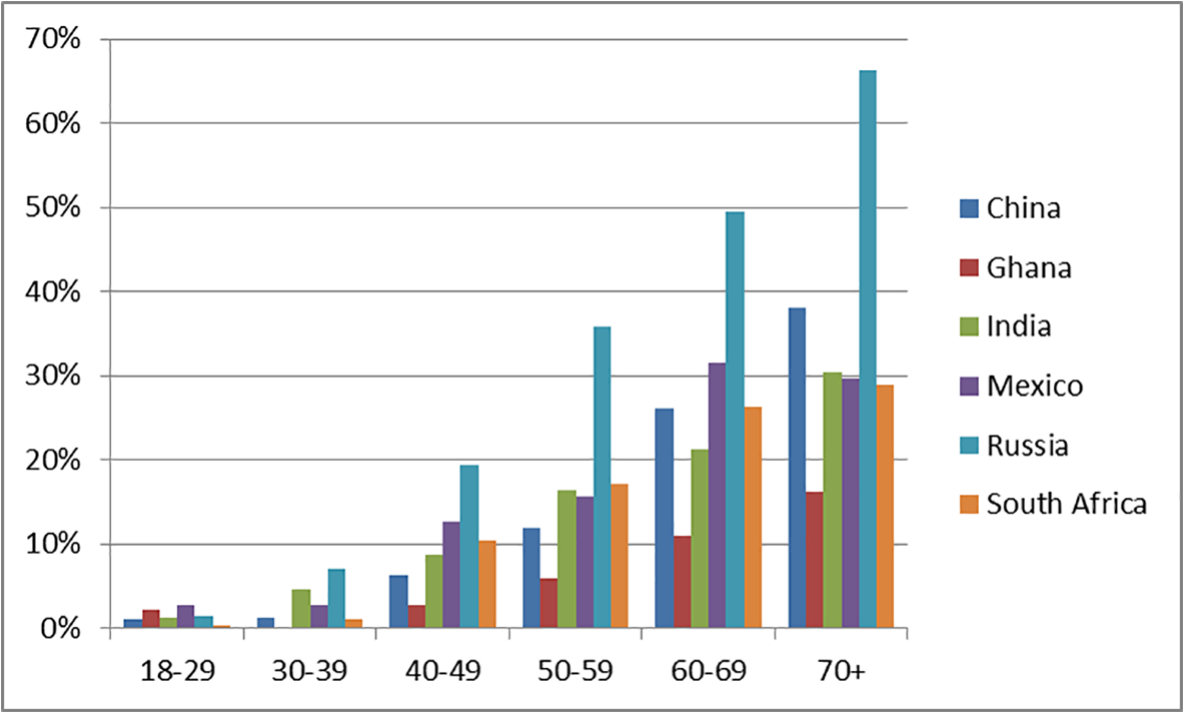
Prevalence of multimorbidity by age groups in SAGE countries.

The prevalence of multimorbidity increased substantially with age in all countries (Table 1). In the pooled countries analysis, prevalence increased from 1.4% in 18–29 year olds to 40.0% in those aged 70+ years (AOR = 45.62, 95% CI = 27.39, 77.99). We observed the most dramatic rise in Russia where the prevalence of multimorbidity increased from 1.5% in 18–29 year olds to 66.2% in year those aged 70+ years. The prevalence of multimorbidity in the 70+ age group was 16.3% in Ghana, 29% in South Africa, 29.6% in Mexico, 30.4% in India, 38.0% in China and 66.2% in Russia (Fig 1). Overall, for the age 50 and above group, 26.8 percent of the population has multimorbidity.

**Table 1:** Socio-demographic correlates of multimorbidity in SAGE countries.

		China	Ghana	India	Mexico	Russia	South Africa	Pooled
		(n = 13,191)	(n = 4,873)	(n = 11,043)	(n = 2,595)	(n = 4,268)	(n = 3,243)	(n = 39,213)
Age	18–29	—	—	—	—	—	—	—
	30–39	1.14 (0.14, 9.36)	—	4.06 (2.15, 7.65)	1.06 (0.25, 4.49)	4.93 (0.54, 45.01)	2.80 (0.18, 42.46)	2.14 (1.22, 3.78)
	40–49	5.94 (1.06, 33.31)	1.43 (0.27, 7.52)	7.82 (4.24, 14.43)	5.46 (1.31, 22.71)	15.61 (2.43, 100.36)	35.41 (3.63, 345.81)	7.08 (4.20, 11.94)
	50–59	11.09 (2.04, 60.28)	3.20 (0.67, 15.21)	16.07 (8.82, 29.27)	5.79 (1.32, 25.29)	38.83 (5.64, 267.60)	60.93 (7.75, 479.01)	14.22 (8.67, 23.33)
	60–69	27.19 (4.79, 154.25)	6.65 (1.42, 31.10)	23.39 (13.05, 41.94)	17.28 (4.34, 68.81)	72.53 (10.15, 518.12)	103.76 (12.53, 859.71)	27.75 (16.71, 46.06)
	70+	41.50 (7.54, 228.36)	10.64 (2.31, 49.10)	39.29 (20.89, 73.89)	14.28 (3.48, 58.57)	153.34 (22.11, 1063.72)	112.75 (13.73, 925.87)	45.62 (27.39, 75.99)
Gender	Male	—	—	—	—	—	—	—
	Female	1.18 (0.85, 1.63)	0.98 (0.60, 1.59)	1.20 (0.97, 1.47)	3.22 (1.89, 5.50)	1.31 (0.88, 1.96)	3.92 (2.37, 6.47)	1.39 (1.17, 1.64)
Location	Rural	—	—	—	—	—	—	—
	Urban	2.24 (1.57, 3.21)	1.41 (0.86, 2.31)	1.05 (0.81, 1.36)	3.73 (1.79, 7.79)	1.60 (1.03, 2.48)	1.62 (0.92, 2.85)	1.55 (1.30, 1.85)
Marital Status	Married	—	—	—	—	—	—	—
	Not Married	0.98 (0.72, 1.32)	1.64 (1.09, 2.48)	0.94 (0.74, 1.20)	1.09 (0.55, 2.16)	1.19 (0.83, 1.69)	0.76 (0.50, 1.16)	1.05 (0.89, 1.23)
Education	No formal	—	—	—	—	—	—	—
	Primary completed	0.58 (0.31, 1.09)	0.54 (0.20, 1.45)	1.44 (1.04, 2.00)	1.08 (0.44, 2.69)	1.51 (0.58, 3.91)	0.81 (0.37, 1.78)	0.94 (0.71, 1.26)
	High school above	0.54 (0.31, 0.93)	0.90 (0.51, 1.59)	1.16 (0.87, 1.55)	0.32 (0.13, 0.78)	1.38 (0.56, 3.37)	0.62 (0.27, 1.43)	0.86 (0.65, 1.14)
Wealth	Q1 (the lowest)	—	—	—	—	—	—	—
	Q2	0.96 (0.64, 1.42)	1.74 (0.94, 3.26)	1.12 (0.73, 1.72)	0.36 (0.14, 0.94)	1.42 (0.79, 2.57)	0.78 (0.32, 1.87)	1.04 (0.82, 1.32)
	Q3	0.84 (0.59, 1.19)	1.69 (1.06, 2.68)	1.28 (0.90, 1.80)	0.87 (0.31, 2.42)	1.51 (0.83, 2.72)	1.11 (0.40, 3.07)	1.20 (0.95, 1.52)
	Q4	0.95 (0.58, 1.55)	5.31 (2.58, 10.93)	1.33 (0.93, 1.91)	0.61 (0.24, 1.56)	1.26 (0.63, 2.51)	1.07 (0.46, 2.52)	1.24 (0.95. 1.63)
	Q5 (the highest)	1.22 (0.70, 2.13)	4.80 (2.83, 8.13)	1.43 (0.99, 2.07)	0.98 (0.38, 2.51)	1.62 (0.95, 2.77)	1.33 (0.57, 3.09)	1.47 (1.15, 1.89)

Notes

1. Multimorbidity defined as two or more chronic conditions in the same individual

2. Additional covariates included in the model in China: provinces; Ghana: ethnic groups (Akan, Ga-Adangbe, and others); India: states; South Africa: provinces, ethnic groups (back, white, coloured, and others).

3. Country dummy variables were included in the model to adjust for heterogeneity among countries in the pooled analysis.

### Socio-demographic correlates of multimorbidity

The prevalence of multimorbidity was higher in urban compared to rural areas (AOR = 1.55, 95% CI = 1.30, 1.85 in the pooled analysis). This association was observed in all countries except India and Ghana. In three countries studied (India, Ghana and Russia), persons in the highest wealth quintile were more likely to have multimorbidity, compared with the poorest (AOR = 1.47, 95% CI = 1.15, 1.89 in pooled analysis).

### Multimorbidity and healthcare utilisation

**Fig 2:**
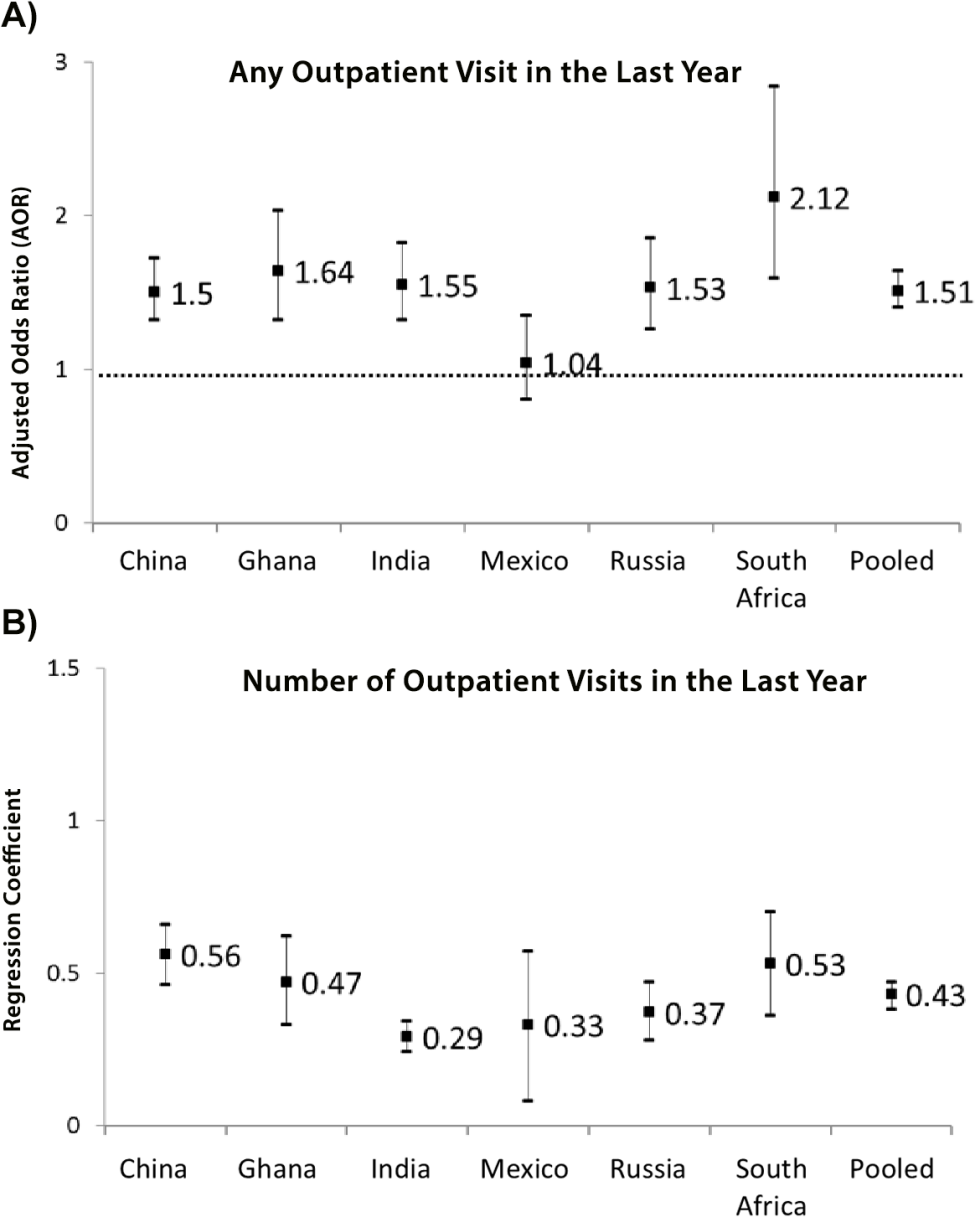
Association between number of NCDs and healthcare utilisation- any outpatient utilisation (Fig 2a); Association between number of NCDs and healthcare utilisation- number of outpatient visits (Fig 2b). Figures in the last column are coefficients and 95% CI for the variable “number of NCD” from regression models adjusting for all covariates. Logistic model is used to estimate any visit for outpatient/inpatient service, and negative binomial model is used for number of visit/ hospitalisation days outcome.

An increased number of NCDs was associated with a higher likelihood of having an outpatient visit in the last year (Table 2, Fig 2). For example, in China, the percentage of respondents having any outpatient visits in the past year increased from 46% for those without any NCDs to 63% for those with more than three NCDs (AOR = 1.50, 95% CI = 1.32,1.72). In South Africa, the percentage of respondents having any outpatient visits increased from 26% for those without any NCD to 81% for those with multimorbidity (AOR = 2.12, 95% CI = 1.59, 2.84). In the pooled analysis, the percentage of respondents having an outpatient visit increased from 51% for those without any NCD to 72% for those with multimorbidity (AOR = 1.51, 95% CI = 1.40, 1.64).

**Table 2:** Association between number of NCDs and healthcare utilisation.

**China**		Zero NCD	One NCD	Multi-Morbidity
Outpatient	Any visit (%)	0.46	0.61	0.63
	Number of visits (mean)	1.14	2.96	4.49
Inpatient	Any hospitalisation	0.09	0.20	0.28
	Hospitalisation days	0.07	0.17	0.23
**Ghana**		Zero NCD	One NCD	Multi-Morbidity
Outpatient	Any visit	0.52	0.65	0.81
	Number of visits	1.28	2.27	3.78
Inpatient	Any hospitalisation	0.11	0.17	0.14
	Hospitalisation days	0.09	0.18	0.14
**India**		Zero NCD	One NCD	Multi-Morbidity
Outpatient	Any visit	0.71	0.84	0.86
	Number of visits	2.23	3.22	5.03
Inpatient	Any hospitalisation	0.09	0.15	0.25
	Hospitalisation days	0.06	0.14	0.23
**Mexico**		Zero NCD	One NCD	Multi-Morbidity
Outpatient	Any visit	0.29	0.35	0.38
	Number of visits	1.38	2.00	2.59
Inpatient	Any hospitalisation	0.09	0.18	0.17
	Hospitalisation days	0.01	0.06	0.20
**Russia**		Zero NCD	One NCD	Multi-Morbidity
Outpatient	Any visit	0.44	0.67	0.72
	Number of visits	0.96	1.51	3.28
Inpatient	Any hospitalisation	0.11	0.22	0.35
	Hospitalisation days	0.08	0.17	0.31
**South Africa**		Zero NCD	One NCD	Multi-Morbidity
Outpatient	Any visit	0.26	0.50	0.81
	Number of visits	0.72	2.63	5.74
Inpatient	Any hospitalisation	0.06	0.09	0.21
	Hospitalisation days	0.10	0.10	0.20
**Pooled**		Zero NCD	One NCD	Multi-Morbidity
Outpatient	Any visit	0.51	0.65	0.72
	Number of visits	1.45	2.66	4.15
Inpatient	Any hospitalisation	0.09	0.18	0.28
	Hospitalisation days	0.07	0.15	0.25

Number of NCDs was associated with increased number of outpatient visits in the last 12 months. For example, in India, the mean number of outpatient visits increased from 2.23 for those without any NCD to 5.03 for those with multimorbidity (coefficient = 0.29, 95% CI = 0.24, 0.34). In Mexico, the mean number of outpatient visits increased from 1.38 for those without any NCD to 2.50 for those with multimorbidity (coefficient = 0.33, 95% CI = 0.08, 0.57). In the pooled analysis, the mean number of outpatient visits increased from 1.45 for those without any NCD to 4.15 for those with multimorbidity (coefficient = 0.43, 95% CI = 0.38, 0.47).

**Fig 3:**
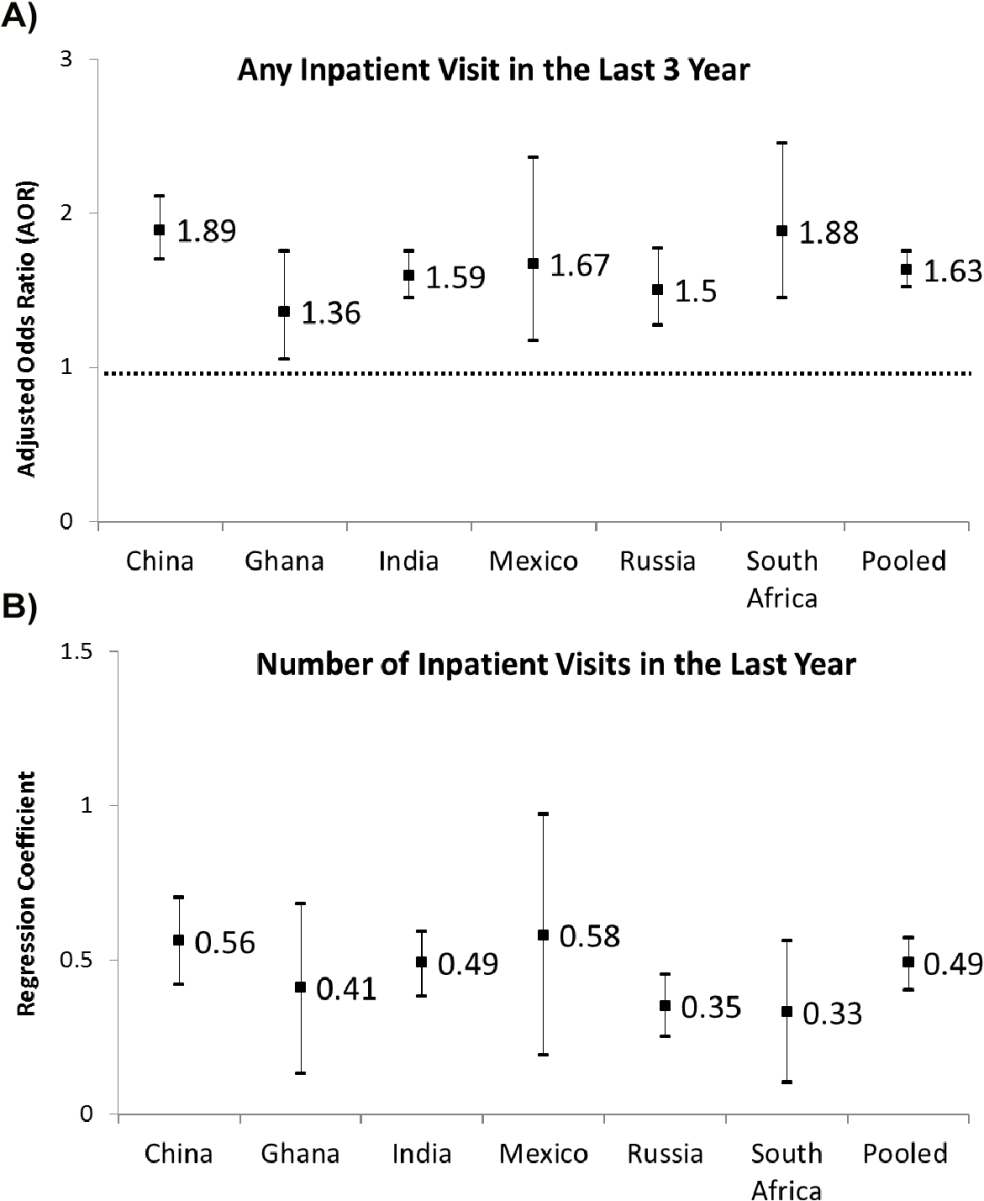
Association between number of NCDs and healthcare utilisation- any inpatient utilisation (Fig 3a); Association between number of NCDs and healthcare utilisation- number of inpatient visits (Fig 3b). Figures in the last column are coefficients and 95% CI for the variable “number of NCD” from regression models adjusting for all covariates. Logistic model is used to estimate any visit for outpatient/inpatient service, and negative binomial model is used for number of visit/ hospitalisation days outcome.

An increased number of NCDs was associated with a higher likelihood of having any hospitalisation over the last three years in all countries (Fig 3). For instance, in Russia, the proportion having any hospital stay increased from 11% for those without any NCDs to 35% for those with more than three NCDs (AOR = 1.53, 95% CI = 1.26, 1.85). In the pooled analysis, the proportion of having any hospital stay increased from 9% for those without any NCDs to 28% for those with NCD multimorbidity (AOR = 1.63, 95% CI = 1.52, 1.75).

An increased number of NCDs was associated with an increased number of hospitalisation days in the last 12 months in all countries. For example, in Ghana, the mean number of hospitalisation days increased from 0.09 for those without any NCDs to 0.14 for those with multimorbidity (coefficient = 0.41, 95% CI = 0.13, 0.68). In the pooled analysis, the mean number of hospitalisation days increased from 0.07 for those without any NCDs to 0.25 for those with multimorbidity (coefficient = 0.49, 95% CI = 0.40, 0.57).

### Multimorbidity and out-of-pocket expenditures

**Fig 4:**
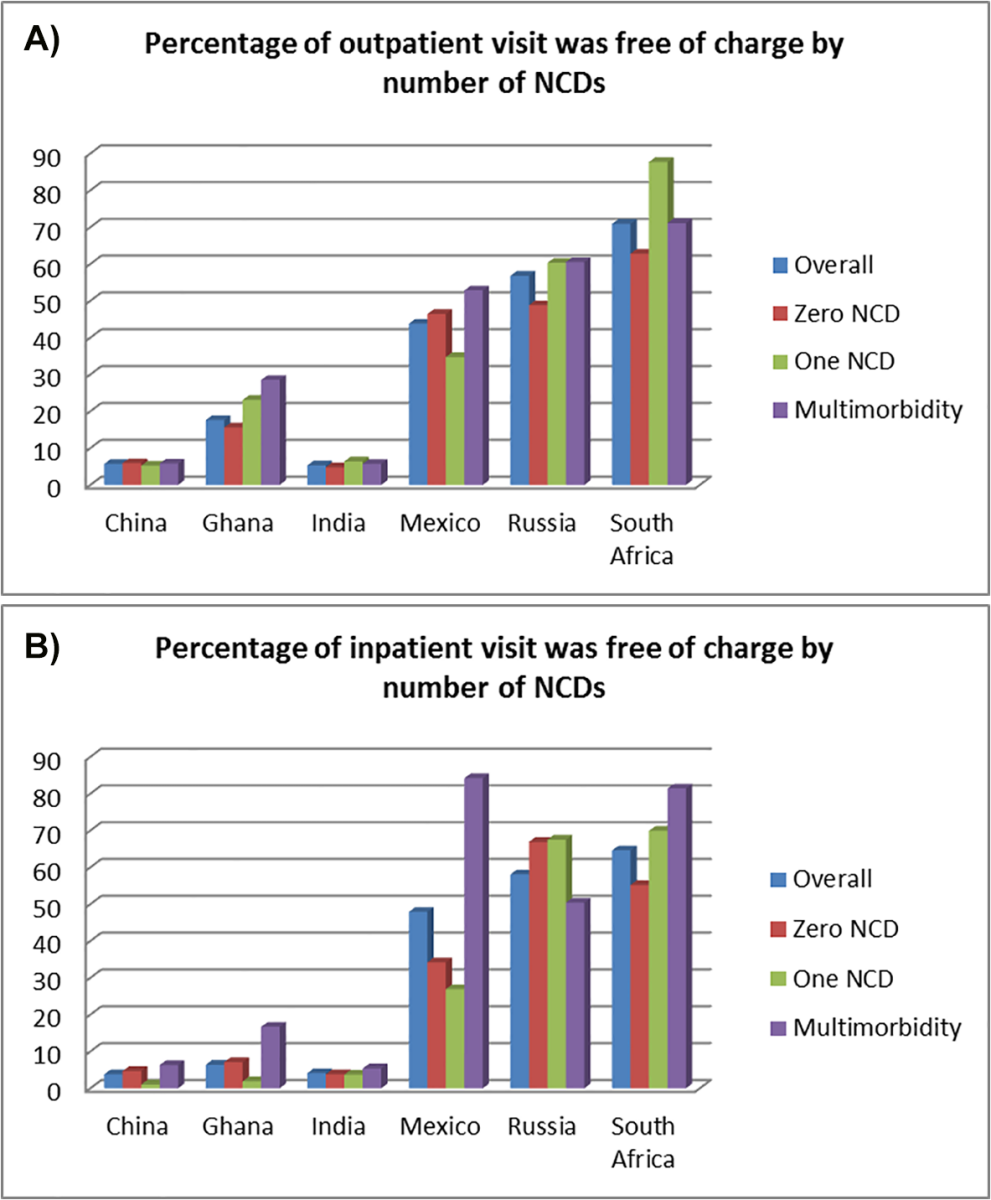
Proportion of respondents reporting that their last outpatient visit was free of charge (Fig 4a); Proportion of respondents reporting that their last inpatient visit was free of charge (Fig 4b). Data source World Health Organization (WHO) Study on Global Ageing and Adult Health (SAGE) survey, wave 1.

The percentage of doctor visits that were free of charge for outpatient and inpatient care are presented in Fig 4A and 4B. Our results suggest less than 5% of respondents who reported that their last health care visit was free in China and India. More than half of doctor visits for outpatient services in Russia and South Africa were free of charge.

Out-of-pocket expenditures incurred in the last visit for outpatient and inpatient services are presented in Table 3. There was a positive association between the number of NCDs and outpatient OOP expenditure in China and India, but the reverse was observed when looking at the inpatient visits in South Africa. In China, mean expenditure for outpatient services in the last visit increased from 124.4 in respondents with no NCD to 179.2 Chinese yuan in respondents with NCD multimorbidity (coefficient = 0.21, 95% CI = 0.12, 0.31). In India, mean OOP expenditure for outpatient service in the last visits increased from 262.2 in respondents with no NCD to 431.0 Indian rupees in respondents with NCD multimorbidity (coefficient = 0.25, 95% CI = 0.18, 0.33). We did not find an increase in inpatient OOP expenditures during the last visit with number of NCDs in any country, except Russia (coefficient = 0.76, 95% CI = 0.37, 1.15). Inpatient OOP expenditure was negatively associated with number of NCDs in South Africa (coefficient = -0.43, 95% CI = -0.79, -0.07).

**Table 3:** Association between number of NCDs and out-of-pocket spending.

**China (in yuan)**	Zero NCD	One NCD	Multimorbidity	Regression Coefficient
Outpatient	124.4	131.6	179.2	0.21 (0.12, 0.31)
Inpatient	4927.0	4160.9	3782.5	-0.08 (-0.28, 0.11)
**Ghana (in cedi)**	Zero NCD	One NCD	Multimorbidity	Regression Coefficient
Outpatient	104915.4	102665.1	80534.5	0.33 (-0.98, 0.31)
Inpatient	606336.4	1023590.0	560583.2	0.09 (-0.76, 0.96)
**India (in rupee)**	Zero NCD	One NCD	Multimorbidity	Regression Coefficient
Outpatient	262.2	340.2	431.0	0.25 (0.18, 0.33)
Inpatient	7653.4	6618.7	6942.1	0.09 (-0.09, 0.26)
**Mexico (in peso)**	Zero NCD	One NCD	Multimorbidity	Regression Coefficient
Outpatient	208.1	183.8	468.8	0.43 (-0.03, 0.88)
Inpatient	3641.7	7346.6	2023.1	0.17 (-0.76, 1.10)
**Russia (in ruble)**	Zero NCD	One NCD	Multimorbidity	Regression Coefficient
Outpatient	500.7	356.7	331.3	-0.02 (-0.25, 0.20)
Inpatient	1012.4	708.8	1558.3	0.76 (0.37, 1.15)
**South Africa (in rand)**	Zero NCD	One NCD	Multimorbidity	Regression Coefficient
Outpatient	53.6	17.2	43.7	-0.24 (-0.46, -0.01)
Inpatient	770.4	5499.0	555.6	-0.43 (-0.79, -0.07)

**Notes:** Figures in the last column are regression coefficients and 95% CI for the variable “number of NCD” from regression models adjusting for all covariates. Log-linear models is used to estimate both outpatient and inpatient out-of-pocket spending outcomes.

Our stratified analyses (Table C in S1 File) indicate that the association between multimorbidity and healthcare utilisation and OOP expenditure were broadly similar across population in different wealth groups, and geographic locations (urban vs rural).

### Source of out-of-pocket expenditures in outpatient and hospital settings

**Fig 5:**
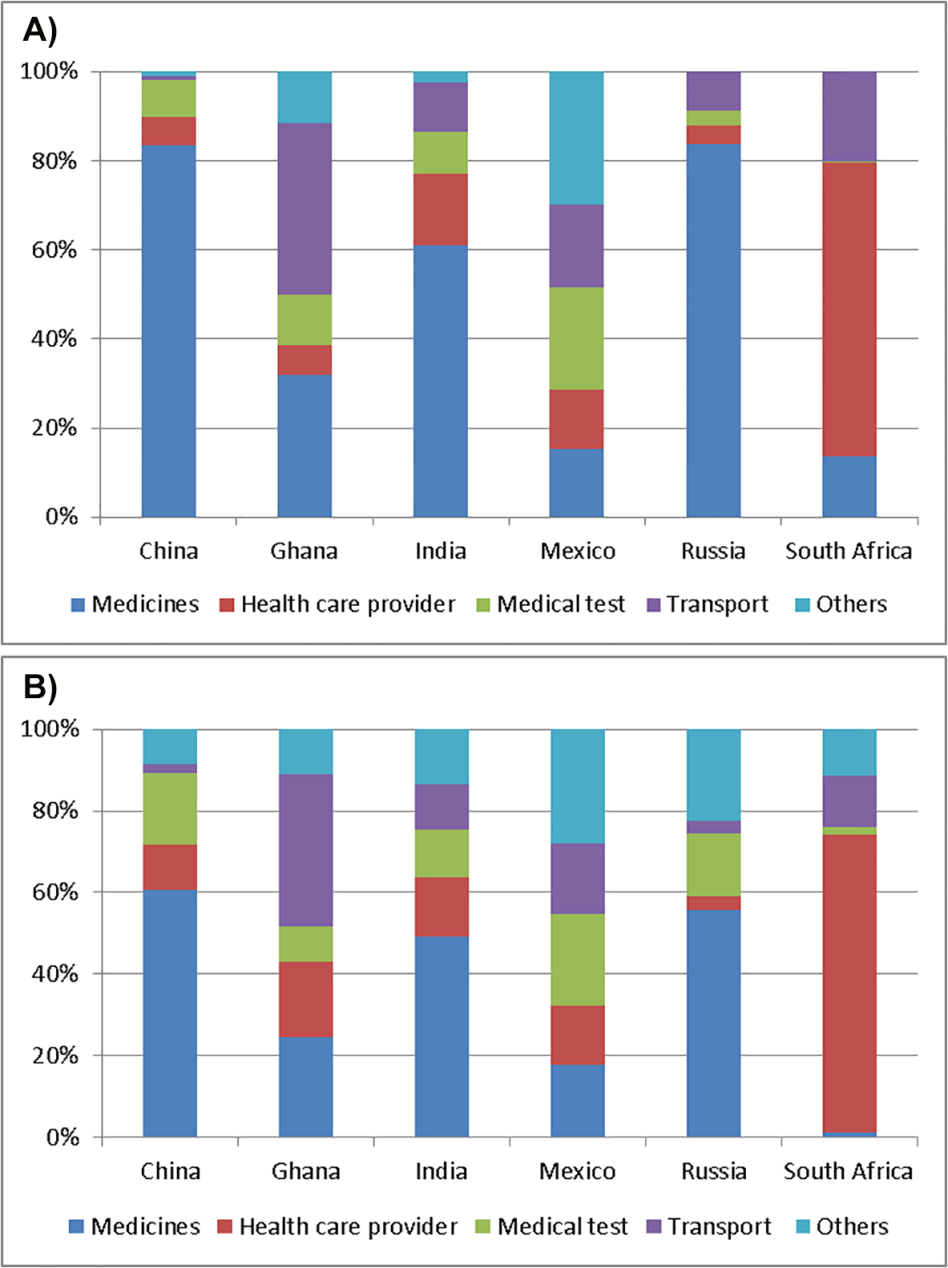
Percentage of out-of-pocket spending for each type of outpatient service in persons with multimorbidity (Fig 5a); Percentage of out-of-pocket spending for each type of inpatient service in persons with multimorbidity (Fig 5b). Estimates presented here are based on respondent’s healthcare visit that was not free of charge.

Patterns of out-of-pocket spending by type of service for persons with multimorbidity are presented in Fig 5 and Tables E and F within S1 File. For outpatient services, medicines constituted the highest proportion of out-of-pocket expenditures for persons with multimorbidity in China (83.5%) and India (61.0%). The proportion of out-of-pocket expenditure for healthcare providers varied greatly between countries, ranging from 4.2% in Russia to 65.9% in South Africa. Transport cost constituted a substantial proportion of spending in Ghana (38.5%), South Africa (20.1%), and Mexico (18.8%). For inpatient services, medicines constituted the highest proportion of out-of-pocket expenditures in China (60.6% of total out-of-pocket expenditures) and India (49.2%). The proportion spent on inpatient healthcare provider fees varied greatly in each country; from 73.2% in South Africa to 3.2% in Russia.

## Discussion

### Main findings

Using nationally representative data from the WHO SAGE, we believe this is the first study to examine the prevalence of multimorbidity in six middle-income countries, its impact on healthcare utilisation and out-of-pocket spending.[29, 36, 37] We found the prevalence of multimorbidity in the adult population to vary markedly, from 3.9% in Ghana to 33.6% in Russia. Consistent with earlier studies, we found that the prevalence of multimorbidity rises substantially with increasing age, and is higher among persons living in urban areas.[5, 8, 38–40] We identified a higher prevalence of multimorbidity among the most affluent groups, which contrasts with some earlier studies from LMICs.[5, 36] This could be due to self-reporting of NCD status in our data, as respondents from higher socioeconomic background have better access to health services resulting in better diagnoses of their NCDs[37].

Our results revealed that NCD multimorbidity is associated with greater healthcare utilisation and greater financial burden for citizens in the countries studied–burden mainly driven by increased healthcare utilisation (for both primary and secondary care), and in some cases by higher out-of-pocket expenditures per visit; findings consistent with earlier studies conducted in high-income settings. For example, using a national representative sample in the United States, Hwang et al.[31] found that mean out-of-pocket spending was nearly five times greater in persons with more than three NCDs compared to those without any NCDs.

Consistent with earlier studies showing the impoverishing effect of purchasing medicines in developing countries[41], our study found spending on medicines constituted a significant proportion of total healthcare expenditures for those with multimorbidity. For example, in China, it constituted 83.5% of outpatient service spending, and 60.6% of inpatient service spending. Our data also shows that a sizeable proportion of out of pocket expenditures were for transport costs, especially in Mexico, Russia, and South Africa.

While there was a high degree of consistency in our country level results, a number of important variations are worth considering. For example, the prevalence of multimorbidity was markedly higher in Russia than in other countries. This could be due to higher disease burden, lower rates of undiagnosed conditions or measurement bias. We found the greater burden of chronic conditions on out-of-pocket spending in countries such as China and India where patients are required to pay a high level of patient cost sharing even for those have health insurance. Whereas, in contrast to other countries, out-of-pocket spending did not increase with more NCDs which reflects that primary care is provided free of charge in South Africa. It is noteworthy that utilization of secondary care followed similar pattern with increasing NCDs as that seen in other countries suggesting that removing financial barriers to accessing primary care may not attenuate the greater risk of hospital admissions in persons with multimorbidity[42].

### Strength and limitations

Our findings are based on a large, nationally representative sample, which provides robust cross-national level estimates of our key variables^27,28^, but several caveats merit discussion. First, the use of survey data and self-reported measures of NCDs are a potential source of bias, including the potential for greater under-reporting of NCDs in persons from lower socioeconomic backgrounds[37, 43, 44]. Although SAGE asks about nine common NCDs, the list is not exhaustive and some common NCDs were not included[39]. Self-reporting of healthcare utilisation and out-of-pocket spending is prone to recall error especially in older populations.[45] There were fewer respondents experienced any hospitalisation over the last year, we have small sample size of less than a hundred observations in some countries when estimating the out-of-pocket spending for their inpatient services. It is warranted for further studies to use administrative or survey dataset with a larger sample to investigate out-of-pocket expenditure made by patients with multimorbidity. Second, SAGE data does not have information on non-medical out-of-pocket expenditures, spending associated with home care and loss of employment income which could be considerable for patients with multiple chronic illnesses.[29, 30] Healthcare utilisation was not specific for NCDs, and may include patient’s visit for communicable and other acute conditions, which may or may not relate to NCDs. Measures for utilisation and expenditure may also not reflect the actual financial burden associated with NCDs, as patients with multiple NCDs may have forgone some treatments because of the anticipated cost related to treatment.[46] Third, we assessed multimorbidity by counting the number of NCDs without applying any weights to account for severity of conditions.[7] Fourth, the cross-sectional study design limits causal interpretation of our findings. As all of these countries have plans to widen health insurance coverage and reduce patient cost sharing, further evidence is needed to assess the causal impact of these policies on healthcare utilisation, medical adherence and patient’s health using longitudinal data.

### Policy implications of findings

Our study provides further evidence for policies and targeted interventions to tackle the growing burden of NCD multimorbidity. Despite the growing prevalence of multimorbidity, current clinical practice frequently emphasise a single-disease specific approach. Our findings suggest more focus should be placed on how best to treat multiple chronic conditions and multiple risks associated with these with a patient-centred approach that fosters greater integration of primary health care based services in health systems.[47] The National Institute for Health and Care Excellence (NICE) in the UK is beginning to develop clinical guideline for multimorbidity, and such efforts are warranted in LMICs where has different health systems with those in high income countries[5, 48]. For example, many LMICs do not have a clear separation of the primary and secondary care as that in the UK, and patients can see specialist without a referral from their general practitioner (GP)[49, 50].

Stronger health systems underpinned by primary health care (PHC) are crucial to effectively manage NCDs and risk factors for them, as PHC is often the first gateway to health services for people with NCDs and plays a central co-ordinating role in the prevention, diagnosis and long-term management of chronic diseases. Clinical guidelines for chronic care and integrated models of care for treating patients with multiple chronic diseases would help to achieve better management of individuals with multimorbidity.[48, 51] Concerted efforts are also needed to improve primary health care not only for treatment of chronic diseases but also to reduce population risk factors for NCDs through intersectoral health promotion and other primary and secondary prevention.[47, 52]

Our results revealed a significant proportion of the sample populations in Mexico (12.6%), Russia (19.4%) and South Africa (10.4%) aged 40–49 years also reported two or more NCDs; this suggests that interventions for those with multimorbidity should not be restricted to older age groups. As most of the NCDs are preventable, more evidence is needed to evaluate the effectiveness and cost-effectiveness of health policies to reduce burden of NCDs such as interventions to improve diet and physical activity[53].

This study has shown greater financial burden for those with NCD multimorbidity in LMICs. Our results can be seen as elasticity of NCDs and healthcare utilization and out-of-pocket spending, and these can be used to populate forecasting models to predict future burden of the NCDs. The forecasting models can further assess the potential impact of preventive policies on NCDs and the economic consequences of these policies[54]. More investment for health and financial innovations as well as affordable health technologies are needed to protect those with chronic diseases[55, 56]. Universal health insurance should be at the centre of policies to promote fair financing and better access to health services across whole populations.[57] Equitable access to effective and safe medicines remains a challenge in LMICs, and issues such as the high cost of branded compared with generic medicines must be addressed to expand access[58]. User fees have detrimental effect on adherence to medicine for patients with chronic conditions, and that there is evidence to suggest that reducing medication co-payments does not lead to an increase in overall health expenditure by governments.[59–61] Further research is required to better understand the additional demands of multimorbidity on health systems and the cost-effectiveness of different strategies to reduce the burden of multimorbidity on individuals and health systems in LMICs.

Our findings supports the WHO call for universal health coverage in LMICs[62], in particular for vulnerable groups such as the elderly who are more likely to have multiple NCDs and incur out-of-pocket expenditures and hence risk impoverishment. Our results have shown that payment for medicines constitute a significant proportion of healthcare spending for those with multimorbidity–with high levels of out-of-pocket expenditures that risk impoverishment. Efforts are needed to lower patient cost sharing for essential and cost-effective treatments to improve financial protection for patients with NCDs and multimorbidity. Decisive action is critical for effective management of NCDs, multimorbidity and the risk factors that determine them if WHO 25x25 targets are to be achieved, vulnerable groups protected from illness-related impoverishment, and the promise of grand convergence in global health outcomes realised.[63, 64]

### Ethics approval

The SAGE study received human subjects testing and ethics council approval from research review boards local to each participating site, and from the WHO Ethical Review Committee, as detailed elsewhere[34]. Informed consent was obtained from each respondent before interview and examination.

## Supporting Information

S1 File Table A in S1 File: Health Statistics for six middle income countries; Figure A in S1 File: Life expectancy and purchasing power parity (PPP) per capita; Figure B in S1 File: Public spending on health as % of GDP and out-of-pocket expenditure as % of total expenditure on health; Table B in S1 FIle: Sample characteristics by number of NCDs; Table C in S1 File: Association between number of NCDs and healthcare utilisation and out-of-pocket spending by residence; Table D in S1 FIle: Association between number of NCDs and healthcare utilisation and out-of-pocket spending by wealth quintile; Table E in S1 File: Percentage of out-of-pocket spending for each type of outpatient service by number of NCDs; Table F in S1 File: Percentage of out-of-pocket spending for each type of inpatient service by number of NCDs (DOCX)
