# Supplementary material for: Impact of Noncommunicable Disease Multimorbidity on Healthcare Utilisation and Out-Of-Pocket Expenditures in Middle-Income Countries: Cross Sectional Analysis
Source: PLoS One. 2015 Jul 8;10(7):e0127199. doi: 10.1371/journal.pone.0127199 (PMC4496037; doi:10.1371/journal.pone.0127199)
Supplement: S1 File — (DOCX) [file pone.0127199.s001.docx]

**Supporting Information S1 File**

**Table A: Health Statistics for six middle income countries**

|  | Life expectancy (years) | Fertility rate | Total expenditure on health as % of GDP | | Government expenditure on health as % of total government expenditure | |
| --- | --- | --- | --- | --- | --- | --- |
|  |  |  | 2000 | 2010 | 2000 | 2010 |
| **China** | 75 | 1.7 | 4.6% | 5.0% | 10.9% | 12.1% |
| **Ghana** | 61 | 4.1 | 4.7% | 5.2% | 8.3% | 12.1% |
| **India** | 66 | 2.6 | 4.3% | 3.7% | 7.4% | 6.8% |
| **Mexico** | 77 | 2.3 | 5.1% | 6.3% | 16.6% | 12.1% |
| **Russia** | 69 | 1.6 | 5.4% | 6.5% | 12.7% | 9.7% |
| **South Africa** | 54 | 2.5 | 8.3% | 8.7% | 10.9% | 12.4% |

Source:

Life expectancy and fertility rate data are figures in 2010, extracted from the World Bank http://data.worldbank.org/.

Other data is from WHO Global Health Observatory Data Repository http://apps.who.int/gho/data/node.main

**Figure A: Life expectancy and purchasing power parity (PPP) per capita**

**
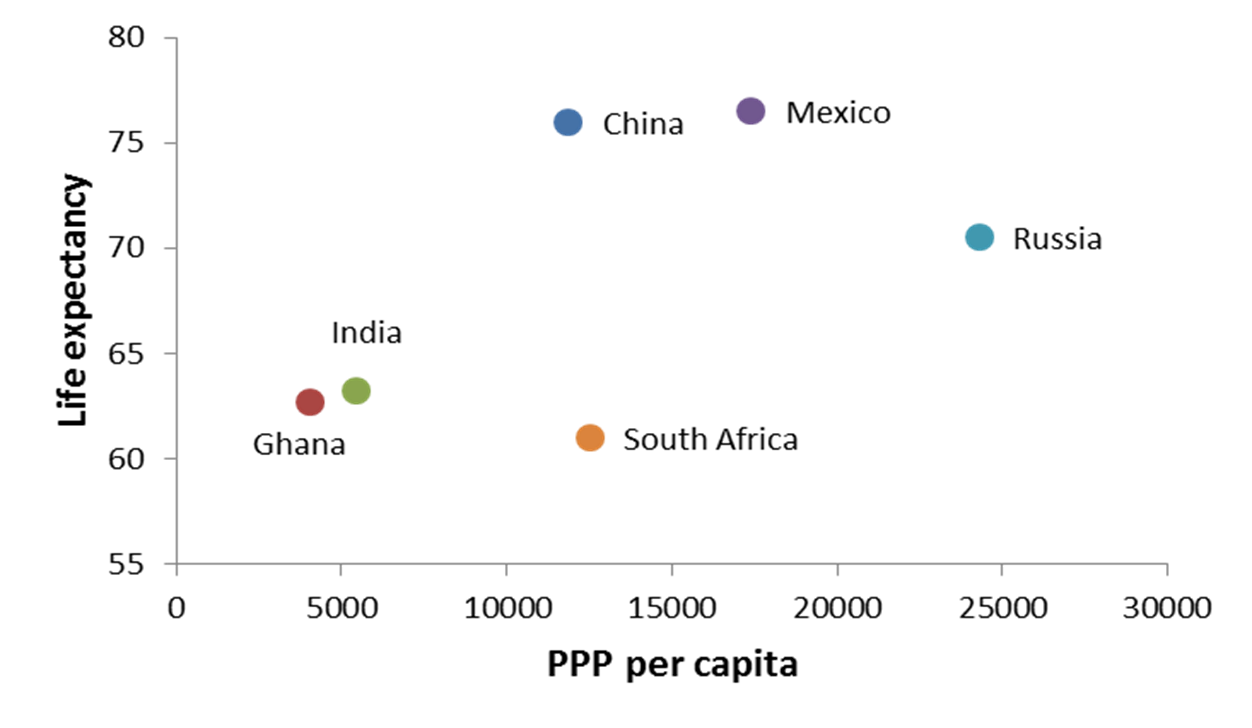
**

**Note: Data from WHO Global Health Observatory (GHO) data repository in year 2012. http://www.who.int/gho/database/en/**

**Figure B: Public spending on health as % of GDP and out-of-pocket expenditure as % of total expenditure on health**

**
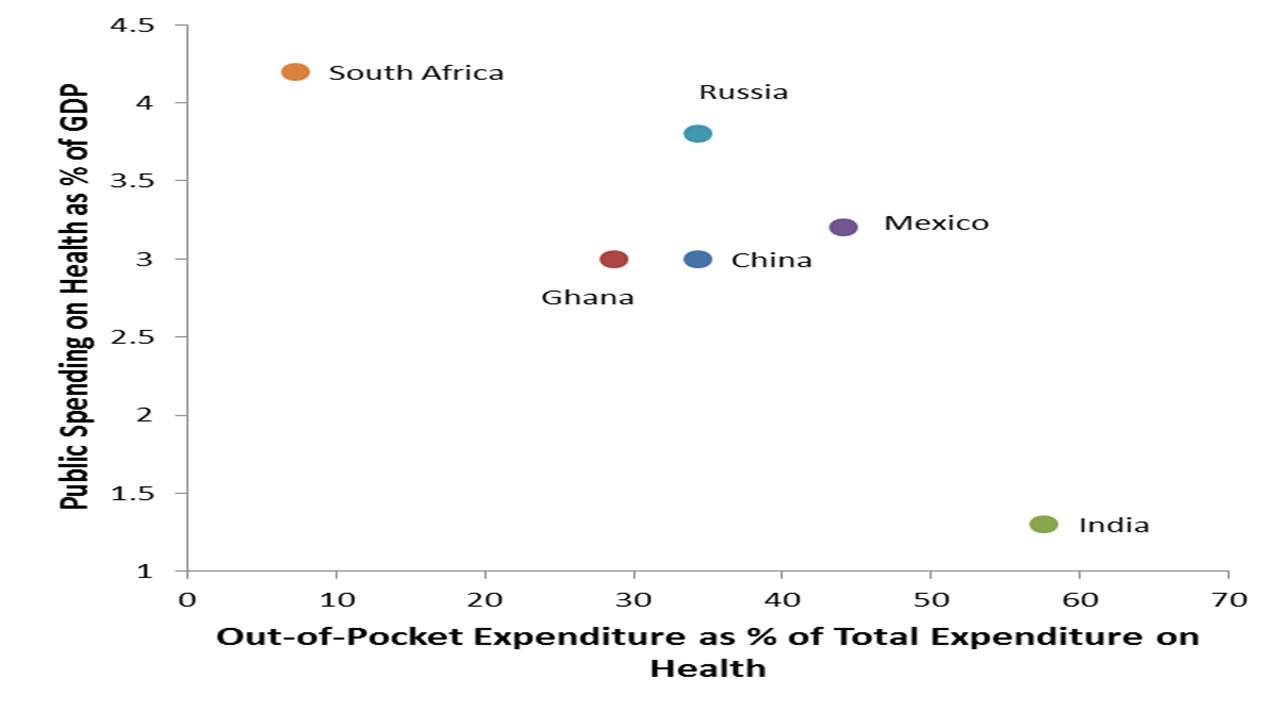
**

**http://www.who.int/gho/database/en/**

**Table B: Sample characteristics by number of NCDs**

| **China** | | Sample size (weighted%) | Number of NCDs (%, 95% CI) | | |
| --- | --- | --- | --- | --- | --- |
|  |  |  | 0 | 1 | 2+ |
| Total population | | 13191 (100) | 71.6 (69.4, 73.7) | 19.4 (17.9, 20.8) | 9.1 (7.8, 10.3) |
| Age | 18-29 | 174 (7.8) | 93.3 (89.1, 97.5) | 5.6 (1.4, 9.9) | 1.1 (-0.6, 2.8) |
|  | 30-39 | 448 (21.1) | 87.9 (84.6, 91.2) | 10.7 (7.3, 14.2) | 1.3 (0.1, 2.6) |
|  | 40-49 | 821 (45.6) | 74.5 (70.9, 78.1) | 19.1 (16.3, 21.9) | 6.4 (3.7, 9.0) |
|  | 50-59 | 4998 (11.2) | 57.1 (54.9, 59.3) | 31.0 (29.0, 33.0) | 11.9 (10.6, 13.2) |
|  | 60-69 | 3558 (8.3) | 41.4 (39.2, 43.6) | 32.4 (30.3, 34.5) | 26.2 (24.4, 28.1) |
|  | 70+ | 3192 (6.0) | 32.1 (30.2, 34.0) | 29.9 (27.8, 31.9) | 38.0 (35.6, 40.5) |
| Gender | Male | 6077 (50.2) | 72.8 (70.2, 75.4) | 19.3 (17.3, 21.3) | 7.9 (6.3, 9.5) |
|  | Female | 7114 (49.8) | 70.3 (67.5, 73.2) | 19.4 (17.2, 21.7) | 10.2 (8.5, 11.9) |
| Residence | Rural | 6563 (50.9) | 73.3 (70.6, 75.9) | 20.0 (18.5, 21.6) | 6.7 (4.9, 8.4) |
|  | Urban | 6628 (49.1) | 69.8 (66.2, 73.5) | 18.7 (16.2, 21.2) | 11.5 (9.7, 13.4) |
| Marital Status | Married | 10991 (89.7) | 71.9 (69.5, 74.2) | 19.5 (17.9, 21.1) | 8.6 (7.3, 10.0) |
|  | Not Married | 2200 (10.3) | 69.1 (64.7, 73.5) | 18.2 (15.3, 21.0) | 12.7 (10.1, 15.4) |
| Education | No formal education | 7763 (37.2) | 63.2 (59.3, 67.1) | 23.0 (20.6, 25.4) | 13.8 (11.2, 16.3) |
|  | Primary school completed | 2851 (32.6) | 76.4 (73.7, 79.1) | 17.9 (15.8, 19.9) | 5.8 (4.0, 7.5) |
|  | High school and above | 2577 (30.3) | 76.7 (73.5, 79.8) | 16.5 (13.4, 19.6) | 6.8 (5.2, 8.5) |
| Wealth | Q1 (the pooest) | 2557 (10.0) | 65.4 (61.2, 69.7) | 22.3 (18.4, 26.1) | 12.3 (10.1, 14.5) |
|  | Q2 | 2546 (15.4) | 73.3 (69.3, 77.3) | 17.9 (14.8, 21.1) | 8.8 (6.8, 10.7) |
|  | Q3 | 2613 (18.0) | 71.7 (66.9, 76.6) | 20.0 (15.7, 24.3) | 8.3 (6.6, 9.9) |
|  | Q4 | 2756 (24.1) | 67.5 (64.2, 70.7) | 24.2 (21.2, 27.3) | 8.3 (5.3, 11.3) |
|  | Q5 (the most affluent) | 2719 (32.6) | 75.6 (70.8, 80.4) | 15.2 (11.9, 18.5) | 9.2 (6.4, 12.1) |

| **Ghana** | | Sample size (weighted%) | Number of NCDs (%, 95% CI) | | |
| --- | --- | --- | --- | --- | --- |
|  |  |  | 0 | 1 | 2+ |
| Total population | | 4873 (100) | 81.9 (79.6, 84.2) | 14.2 (12.1, 16.2) | 3.9 (3.0, 4.9) |
| Age | 18-29 | 123 (12.3) | 92.1 (86.8, 97.4) | 5.7 (1.5, 9.9) | 2.2 (-1.2, 5.5) |
|  | 30-39 | 291 (27.4) | 91.0 (87.1, 94.9) | 9.0 (5.1, 12.9) | -- |
|  | 40-49 | 351 (35.7) | 82.6 (77.8, 87.4) | 14.6 (10.2, 19.0) | 2.8 (0.7, 5.0) |
|  | 50-59 | 1616 (9.8) | 73.9 (70.8, 76.9) | 20.2 (17.6, 22.7) | 6.0 (4.4, 7.5) |
|  | 60-69 | 1157 (6.8) | 65.0 (60.9, 69.2) | 23.9 (20.8, 27.1) | 11.0 (8.7, 13.4) |
|  | 70+ | 1335 (8.0) | 56.2 (52.1, 60.3) | 27.5 (24.5, 30.5) | 16.3 (13.2, 19.3) |
| Gender | Male | 2557 (50.1) | 84.9 (81.8, 87.9) | 11.4 (8.9, 13.8) | 3.8 (2.3. 5.2) |
|  | Female | 2316 (49.9) | 78.9 (75.3, 82.5) | 17.0 (13.5, 20.4) | 4.1 (2.9, 5.3) |
| Residence | Rural | 2881 (54.5) | 85.2 (82.3, 88.1) | 11.9 (9.2, 14.6) | 2.9 (2.0, 3.8) |
|  | Urban | 1992 (45.5) | 77.9 (74.3, 81.6) | 16.9 (13.8, 20.0) | 5.1 (3.4, 7.0) |
| Marital Status | Married | 2828 (69.7) | 84.9 (82.3, 87.6) | 12.1 (9.7, 14.5) | 3.0 (1.8, 4.1) |
|  | Not Married | 2045 (30.3) | 75.0 (70.8, 79.2) | 18.9 (14.9, 22.9) | 6.1 (4.3, 8.0) |
| Education | No formal education | 3580 (63.4) | 82.6 (79.8, 85.5) | 13.2 (10.6, 15.8) | 4.2 (2.9, 5.4) |
|  | Primary school completed | 270 (10.6) | 84.2 (77.4, 90.9) | 13.8 (7.2, 20.5) | 2.0 (0.5, 3.4) |
|  | High school and above | 1023 (26.1) | 79.2 (74.1, 84.3) | 16.7 (12.0, 21.4) | 4.1 (2.0, 6.2) |
| Wealth | Q1 (the pooest) | 946 (15.0) | 86.9 (82.5, 91.4) | 11.5 (7.1, 15.8) | 1.6 (1.0, 2.2) |
|  | Q2 | 963 (17.8) | 87.8 (84.0, 91.6) | 9.5 (6.1, 12.9) | 2.7 (1.4, 4.0) |
|  | Q3 | 964 (19.1) | 82.2 (77.1, 87.2) | 15.4 (10.5, 20.3) | 2.4 (1.6, 3.2) |
|  | Q4 | 999 (22.8) | 79.7 (74.8, 84.7) | 14.5 (10.2, 18.8) | 5.8 (3.0, 8.5) |
|  | Q5 (the most affluent) | 1001 (25.3) | 76.5 (71.0, 82.0) | 17.9 (13.0, 22.8) | 5.6 (3.4, 7.8) |

| **India** | | Sample size (weighted %) | Number of NCDs (%, 95% CI) | | |
| --- | --- | --- | --- | --- | --- |
|  |  |  | 0 | 1 | 2+ |
| Total population | | 11043 (100) | 71.6 (69.8, 73.3) | 19.5 (18.2, 20.9) | 8.9 (8.0, 9.8) |
| Age | 18-29 | 1579 (24.0) | 89.8 (87.7, 92.0) | 8.9 (6.7, 11.0) | 1.3 (0.6, 1.9) |
|  | 30-39 | 1633 (25.2) | 78.5 (75.6, 81.4) | 16.8 (14.1, 19.5) | 4.7 (3.4, 6.1) |
|  | 40-49 | 1387 (25.9) | 68.2 (64.5,71.8) | 23.2 (20.0, 26.3) | 8.7 (6.5, 10.9) |
|  | 50-59 | 2887 (12.1) | 57.2 (54.4, 60.0) | 26.5 (23.9, 29.1) | 16.4 (13.9, 18.8) |
|  | 60-69 | 2193 (7.6) | 47.1 (43.8, 50.5) | 31.7 (28.2, 35.1) | 21.2 (18.4, 24.0) |
|  | 70+ | 1364 (5.1) | 39.0 (34.3, 43.7) | 30.6 (26.9, 34.3 | 30.4 (25.5, 35.3) |
| Gender | Male | 4266 (50.8) | 71.8 (69.1, 74.6) | 19.4 (17.2, 21.6) | 8.8 (7.4, 10.1) |
|  | Female | 6781 (49.2) | 71.3 (69.5, 73.1) | 19.7 (18.0, 21.4) | 9.0 (8.0, 10.0) |
| Residence | Rural | 8242 (74.5) | 72.5 (70.6, 74.3) | 19.3 (17.8, 20.8) | 8.3 (7.3, 9.3) |
|  | Urban | 2801 (25.5) | 69.0 (65.2, 72.7) | 20.4 (17.5, 23.2) | 10.7 (8.7, 12.6) |
| Marital Status | Married | 8575 (82.0) | 71.6 (69.8, 73.3) | 19.6 (18.2, 21.1) | 8.8 (7.8, 9.8) |
|  | Not Married | 2468 (18.0) | 71.5 (68.1, 74.9) | 19.1 (16.3, 21.9) | 9.4 (7.8, 10.9) |
| Education | No formal education | 7856 (61.6) | 70.1 (68.2, 71.9) | 20.4 (18.9, 21.9) | 9.6 (8.6, 10.6) |
|  | Primary school completed | 1375 (15.7) | 74.5 (70.8,78.3) | 17.1 (14.0, 20.2) | 8.3 (6.0, 10.7) |
|  | High school and above | 1812 (22.7) | 73.6 (69.9, 77.3) | 18.9 (15.7, 22.2) | 7.4 (5.6, 9.3) |
| Wealth | Q1 (the pooest) | 1982 (20.8) | 74.5 (71.8, 78.2) | 18.3 (15.6, 21.0) | 6.7 (4.9, 8.5) |
|  | Q2 | 2133 (21.4) | 75.1 (72.2, 78.0) | 17.2 (14.5, 19.9) | 7.7 (5.7, 9.8) |
|  | Q3 | 2112 (19.8) | 70.5 (66.7, 74.4) | 20.5 (17.3, 23.6) | 9.0 (7.1, 10.9) |
|  | Q4 | 2322 (18.0) | 68.4 (65.0, 71.9) | 20.9 (17.7, 24.2) | 10.6 (8.6, 12.7) |
|  | Q5 (the most affluent) | 2494 (20.1) | 68.1 (65.0, 71.3) | 21.2 (18.4, 24.0) | 10.7 (8.9, 12.4) |

| **Mexico** | | Sample size (weighted%) | Number of NCDs (%, 95% CI) | | |
| --- | --- | --- | --- | --- | --- |
|  |  |  | 0 | 1 | 2+ |
| Total population | | 2595 (100) | 64.6 (57.7, 71.5) | 25.1 (18.2, 32.1) | 10.3 (7.9, 12.7) |
| Age | 18-29 | 56 (16.2) | 86.7 (76.2, 97.2) | 10.6 (1.0, 20.2) | 2.7 (-0.6, 5.9) |
|  | 30-39 | 169 (35.4) | 73.4 (55.8, 91.0) | 23.9 (5.8, 41.9) | 2.7 (0.5, 5.0) |
|  | 40-49 | 188 (22.0) | 60.1 (48.3, 71.9) | 27.3 (18.0, 36.6) | 12.6 (4.5, 20.7) |
|  | 50-59 | 422 (12.9) | 56.2 (45.9, 66.4) | 28.2 (17.5, 39.0) | 15.6 (8.4, 22.7) |
|  | 60-69 | 908 (6.8) | 30.6 (25.1, 36.0) | 37.8 (30.9, 44.6) | 31.6 (23.5, 39.7) |
|  | 70+ | 852 (6.6) | 29.1 (23.3, 35.0) | 41.2 (33.2, 49.3) | 29.6 (23.0, 36.3) |
| Gender | Male | 993 (47.2) | 77.7 (70.9, 84.5) | 16.9 (10.7, 23.0) | 5.4 (3.2, 7.7) |
|  | Female | 1602 (52.8) | 52.8 (43.3, 62.4) | 32.5 (21.7, 43.3) | 14.7 (10.4, 18.9) |
| Residence | Rural | 695 (22.8) | 70.9 (60.6, 81.2) | 24.0 (15.6, 32.5) | 5.1 (2.2, 7.9) |
|  | Urban | 1900 (77.2) | 62.7 (54.4, 71.0) | 25.5 (16.8, 34.1) | 11.8 (8.9, 14.8) |
| Marital Status | Married | 1525 (60.6) | 59.4 (50.0, 68.8) | 30.4 (20.4, 40.3) | 10.2 (6.7, 13.6) |
|  | Not Married | 1070 (39.4) | 72.5 (65.6, 79.5) | 17.0 (10.7, 23.4) | 10.4 (6.9, 14.0) |
| Education | No formal education | 1988 (51.7) | 54.5 (44.4, 64.6) | 31.7 (20.7, 42.6) | 13.8 (9.7, 17.9) |
|  | Primary school completed | 277 (23.6) | 74.7 (64.7, 84.7) | 15.7 (8.9, 22.5) | 9.6 (2.9, 16.3) |
|  | High school and above | 330 (24.7) | 75.9 (65.8, 86.1) | 20.5 (10.8, 30.1) | 3.6 (0.9, 6.2) |
| Wealth | Q1 (the pooest) | 538 (16.7) | 70.6 (58.0, 83.1) | 18.4 (9.5, 27.3) | 11.1 (4.3, 17.8) |
|  | Q2 | 534 (23.5) | 64.8 (40.7, 88.9) | 28.9 (4.0, 53.8) | 6.3 (2.1, 10.5) |
|  | Q3 | 481 (20.3) | 69.7 (57.2, 82.3) | 18.0 (8.1, 27.9) | 12.2 (5.1, 19.3) |
|  | Q4 | 535 (14.5) | 50.2 (37.3, 63.0) | 39.4 (26.6, 52.2) | 10.5 (6.2, 14.7) |
|  | Q5 (the most affluent) | 507 (25.0) | 64.6 (53.4, 75.9) | 23.5 (13.7, 33.3) | 11.9 (6.5, 17.3) |

| **Russia** | | Sample size (weighted%) | Number of NCDs (%, 95% CI) | | |
| --- | --- | --- | --- | --- | --- |
|  |  |  | 0 | 1 | 2+ |
| Total population | | 4268 (100) | 41.2 (36.1, 46.3) | 25.2 (21.6, 28.8) | 33.6 (28.8, 38.5) |
| Age | 18-29 | 97 (10.2) | 84.2 (72.0, 96.4) | 14.3 (1.4, 27.1) | 1.5 (-1.3, 4.4) |
|  | 30-39 | 144 (12.6) | 66.6 (56.4, 76.8) | 26.4 (16.2, 36.5) | 7.0 (0.7, 13.3) |
|  | 40-49 | 171 (16.2) | 50.8 (38.3, 63.4) | 29.8 (16.8, 42.8) | 19.4 (9.2, 29.6) |
|  | 50-59 | 1443 (27.0) | 35.9 (29.2, 42.5) | 28.2 (23.4, 33.0) | 35.9 (30.4, 41.4) |
|  | 60-69 | 1052 (16.3) | 23.8 (15.9, 31.7) | 26.8 (17.0, 36.6) | 49.4 (38.5, 60.2) |
|  | 70+ | 1361 (17.6) | 13.6 (7.2, 19.9) | 20.3 (13.1, 27.4) | 66.2 (59.1, 73.3) |
| Gender | Male | 1519 (42.7) | 46.1 (39.4, 52.9) | 25.1 (20.0, 30.2) | 28.8 (21.4, 36.1) |
|  | Female | 2749 (57.3) | 37.5 (31.4, 43.7) | 25.2 (20.5, 29.9) | 37.2 (32.1, 42.4) |
| Residence | Rural | 1041 (24.4) | 39.5 (31.9, 47.2) | 26.1 (19.8, 32.5) | 34.3 (26.1, 42.6) |
|  | Urban | 3227 (75.6) | 41.7 (35.6, 47.8) | 24.9 (20.6, 29.2) | 33.4 (27.5, 39.3) |
| Marital Status | Married | 2294 (56.0) | 42.1 (36.1, 48.1) | 26.7 (21.7, 31.7) | 31.2 (25.5, 36.9) |
|  | Not Married | 1974 (44.0) | 40.0 (33.1, 46.9) | 23.3 (18.4, 28.1) | 36.7 (30.2, 43.2) |
| Education | No formal education | 423 (4.3) | 11.6 (4.7, 18.4) | 32.8 (13.1, 52.5) | 55.7 (37.4, 73.9) |
|  | Primary school completed | 768 (13.2) | 26.4 (16.4, 36.5) | 25.0 (16.7, 33.4) | 48.6 (38.8, 58.3) |
|  | High school and above | 3077 (82.5) | 45.1 (39.1, 51.1) | 24.8 (20.2, 29.4) | 30.1 (24.7, 35.4) |
| Wealth | Q1 (the pooest) | 760 (12.2) | 47.7 (36.7, 58.7) | 18.2 (12.4, 24.0) | 34.1 (26.3, 41.9) |
|  | Q2 | 821 (15.0) | 32.8 (24.6, 41.0) | 26.8 (18.7, 34.9) | 40.4 (31.3, 49.6) |
|  | Q3 | 851 (17.9) | 38.5 (25.7, 51.3) | 22.6 (14.5, 30.6) | 38.9 (28.1, 49.9) |
|  | Q4 | 875 (23.8) | 39.1 (31.2, 47.0) | 32.8 (23.0, 42.5) | 28.1 (20.0, 36.2) |
|  | Q5 (the most affluent) | 961 (31.0) | 45.9 (37.5, 54.3) | 22.8 (16.9, 28.7) | 31.3 (24.6, 37.9) |

| **South Africa** | | Sample size (weighted%) | Number of NCDs (%, 95% CI) | | |
| --- | --- | --- | --- | --- | --- |
|  |  |  | 0 | 1 | 2+ |
| Total population | | 3243 (100) | 73.1 (66.3, 80.0) | 17.7 (11.2, 24.2) | 9.2 (6.7, 11.7) |
| Age | 18-29 | 69 (19.9) | 89.8 (77.7, 102.0) | 9.8 (-2.4, 21.9) | 0.4 (-0.4, 1.2) |
|  | 30-39 | 82 (24.4) | 95.6 (92.0, 99.3) | 3.4 (0.2, 6.5) | 1.0 (-0.6, 2.7) |
|  | 40-49 | 106 (30.3) | 65.3 (47.4, 83.0) | 24.3 (6.4, 42.2) | 10.4 (3.2, 17.5) |
|  | 50-59 | 1307 (12.6) | 53.3 (48.8, 57.9) | 29.5 (25.7, 33.3) | 17.2 (13.9, 20.5) |
|  | 60-69 | 955 (7.7) | 41.8 (36.0, 47.6) | 31.8 (26.9, 36.8) | 26.4 (21.3, 31.4) |
|  | 70+ | 724 (5.1) | 44.0 (38.0, 50.0) | 26.9 (21.2, 32.6) | 29.0 (23.2, 34.9) |
| Gender | Male | 1281 (45.9) | 83.7 (75.0, 92.3) | 12.4 (4.1, 20.8) | 3.9 (2.5, 5.3) |
|  | Female | 1962 (54.1) | 64.2 (54.5, 73.9) | 22.1 (12.7, 31.6) | 13.6 (9.2, 18.1) |
| Residence | Rural | 1074 (26.3) | 80.1 (74.6, 85.6) | 11.7 (7.7, 15.6) | 8.2 (5.0, 11.4) |
|  | Urban | 2169 (73.7) | 70.7 (61.6, 79.7) | 19.8 (11.2, 28.4) | 9.5 (6.3, 12.8) |
| Marital Status | Married | 1436 (36.4) | 68.5 (57.3, 79.7) | 19.9 (9.7, 30.1) | 11.6 (6.6, 16.6) |
|  | Not Married | 1807 (63.6) | 75.8 (67.1, 84.4) | 16.4 (8.0, 24.8) | 7.8 (5.0, 10.5) |
| Education | No formal education | 2324 (37.2) | 62.3 (54.0, 70.5) | 23.1 (15.3, 30.8) | 14.7 (11.0, 18.4) |
|  | Primary school completed | 476 (26.8) | 67.6 (49.1, 85.9) | 26.2 (7.4, 44.9) | 6.3 (2.4, 10.1) |
|  | High school and above | 443 (36.1) | 88.5 (82.7, 94.3) | 5.9 (2.5, 9.2) | 5.7 (1.1, 10.2) |
| Wealth | Q1 (the pooest) | 619 (18.1) | 69.2 (56.0, 82.5) | 22.9 (10.3, 35.6) | 7.9 (2.4, 13.4) |
|  | Q2 | 658 (18.9) | 72.1 (52.5, 91.8) | 21.4 (1.1, 41.7) | 6.5 (3.2, 9.7) |
|  | Q3 | 656 (24.6) | 79.8 (68.2, 91.4) | 12.1 (2.4, 21.8) | 8.1 (3.4, 12.9) |
|  | Q4 | 663 (20.2) | 69.6 (52.6, 86.7) | 20.9 (3.7, 38.1) | 9.5 (4.5, 14.5) |
|  | Q5 (the most affluent) | 647 (18.1) | 73.0 (63.1, 83.0) | 12.6 (6.5, 18.7) | 14.4 (6.8, 21.9) |

**Table C: Association between number of NCDs and healthcare utilisation and out-of-pocket spending by residence**

|  | **China** | **Ghana** | **India** | **Mexico** | **Russia** | **South Africa** |
| --- | --- | --- | --- | --- | --- | --- |
| **Any outpatient visit** | | | | | | |
| Rural | 1.49 (1.27, 1.75) | 1.25 (0.93, 1.70) | 1.41 (1.17, 1.69) | 0.81 (0.44, 1.49) | 2.04 (1.27, 3.27) | 2.79 (1.85, 4.21) |
| Urban | 1.60 (1.30, 1.96) | 2.02 (1.48, 2.75) | 2.11 (1.62, 2.75) | 1.10 (0.83, 1.47) | 1.39 (1.15, 1.67) | 1.99 (1.39, 2.86) |
| **Number of outpatient visits** | | | | | | |
| Rural | 0.50 (0.39, 0.61) | 0.21 (0.03, 0.38) | 0.28 (0.22, 0.34) | 0.31 (-0.46, 1.07) | 0.56 (0.37, 0.75) | 0.82 (0.55, 1.08) |
| Urban | 0.62 (0.48, 0.76) | 0.61 (0.42, 0.81) | 0.31 (0.24, 0.37) | 0.32 (0.07, 0.57) | 0.32 (0.22, 0.42) | 0.47 (0.30, 0.64) |
| **Outpatient out-of-pocket spending** | | | | | | |
| Rural | 0.10 (-0.03, 0.22) | 0.12 (-0.43, 0.68) | 0.25 (0.16, 0.35) | -0.21 (-1.08, 0.65) | -0.05 (-0.36, 0.27) | -0.02 (-0.37, 0.32) |
| Urban | 0.30 (0.15, 0.45) | -0.71 (-1.65, 0.22) | 0.25 (0.12, 0.38) | 0.58 (0.12, 1.03) | -0.04 (-0.31, 0.24) | -0.27 (-0.48, -0.05) |
| **Any inpatient visit** | | | | | | |
| Rural | 1.96 (0.18, 2.29) | 0.93 (0.63, 1.39) | 1.66 (1.49, 1.85) | 2.54 (1.18, 5.47) | 1.78 (1.20, 2.66) | 2.24 (1.42, 3.53) |
| Urban | 1.81 (1.59, 2.06) | 1.62 (1.15, 2.29) | 1.47 (1.24, 1.74) | 1.53 (1.04, 2.26) | 1.41 (1.17, 1.71) | 2.06 (1.48, 2.86) |
| **Hospitalisation days** | | | | | | |
| Rural | 0.59 (0.41, 0.78) | 0.23 (-0.11, 0.57) | 0.54 (0.43, 0.65) | 1.83 (0.68, 2.99) | 0.50 (0.32, 0.69) | 0.50 (0.12, 0.88) |
| Urban | 0.57 (0.40, 0.74) | 0.49 (0.14, 0.84) | 0.38 (0.21, 0.56) | 0.34 (-0.02, 0.71) | 0.31 (0.20, 0.41) | 0.43 (0.17, 0.70) |
| **Inpatient out-of-pocket spending** | | | | | | |
| Rural | 0.06 (-0.17, 0.29) | 1.51 (0.39, 2.63) | 0.06 (-0.14, 0.26) | 1.36 (-0.30, 3.03) | 0.38 (-0.29, 1.06) | 0.01 (-0.62, 0.64) |
| Urban | -0.18 (-0.47, 0.11) | -0.62 (-1.60, 0.35) | 0.04 (-0.24, 0.32) | 0.57 (-0.50, 1.63) | 0.68 (0.26, 1.09) | -0.24 (-0.60, 0.13) |

**Table D: Association between number of NCDs and healthcare utilisation and out-of-pocket spending by wealth quintile**

|  | **China** | **Ghana** | **India** | **Mexico** | **Russia** | **South Africa** |
| --- | --- | --- | --- | --- | --- | --- |
| **Any outpatient visit** | | | | | | |
| Wealth Quintile 1 | 1.46 (1.23, 1.74) | 1.27 (0.65, 2.47) | 1.60 (1.21, 2.12) | 0.40 (-0.24, 1.02) | 2.03 (1.55, 2.63) | 1.78 (1.00, 3.14) |
| Wealth Quintile 2 | 1.69 (1.28, 2.23) | 1.17 (0.72, 1.91) | 1.31 (0.88, 1.95) | 0.41 (-0.39, 1.21) | 1.40 (0.95, 2.06) | 2.92 (1.75, 4.88) |
| Wealth Quintile 3 | 1.54 (1.16, 2.03) | 0.99 (0.65, 1.55) | 1.97 (1.48, 2.63) | 0.49 (0.08, 0.90) | 1.40 (1.06, 1.87) | 5.00 (2.30, 10.89) |
| Wealth Quintile 4 | 1.51 (1.10, 2.06) | 1.54 (1.01, 2.37) | 1.43 (1.03, 1.99) | 0.35 (-0.13, 0.82) | 1.73 (1.18, 2.54) | 2.07 (1.31, 3.25) |
| Wealth Quintile 5 | 1.51 (1.13, 2.02) | 2.45 (1.52, 3.95) | 1.60 (1.21, 2.11) | 0.20 (-0.22, 0.63) | 1.28 (0.90, 1.83) | 2.69 (1.55, 4.69) |
| **Number of outpatient visits** | | | | | | |
| Wealth Quintile 1 | 0.57 (0.42, 0.72) | 0.17 (-0.07, 0.40) | 0.29 (0.22, 0.37) | 0.39 (-0.24, 1.02) | 0.42 (0.28, 0.55) | 0.50 (0.20, 0.81) |
| Wealth Quintile 2 | 0.59 (0.42, 0.75) | 0.11 (-0.21, 0.43) | 0.23 (0.11, 0.34) | 0.41 (-0.39, 1.21) | 0.44 (0.25, 0.63) | 0.53 (0.27, 0.79) |
| Wealth Quintile 3 | 0.53 (0.36, 0.70) | 0.12 (-0.11, 0.34) | 0.33 (0.24, 0.42) | 0.49 (0.08, 0.90) | 0.43 (0.20, 0.66) | 0.85 (0.53, 1.17) |
| Wealth Quintile 4 | 0.49 (0.33, 0.65) | 0.53 (0.30, 0.75) | 0.20 (0.09, 0.31) | 0.35 (-0.13, 0.82) | 0.30 (0.08, 0.52) | 0.40 (0.14, 0.66) |
| Wealth Quintile 5 | 0.55 (0.39, 0.72) | 0.64 (0.36, 0.92) | 0.35 (0.25, 0.45) | 0.20 (-0.22, 0.63) | 0.34 (0.19, 0.48) | 0.48 (0.28, 0.68) |
| **Outpatient out-of-pocket spending** | | | | | | |
| Wealth Quintile 1 | 0.23 (-0.01, 0.47) | 0.36 (-0.37, 1.10) | 0.27 (0.10, 0.44) | -0.09 (-1.07, 0.89) | -0.04 (-0.42, 0.33) | 0.17 (-0.11, 0.44) |
| Wealth Quintile 2 | 0.11 (-0.10, 0.31) | 0.36 (-0.88, 1.60) | 0.29 (0.15, 0.43) | -0.18 (-0.84, 0.47) | 0.12 (-0.26, 0.49) | 0.01 (-0.27, 0.29) |
| Wealth Quintile 3 | -0.05 (-0.21, 0.11) | -0.01 (-1.01, 0.99) | 0.20 (0.07, 0.34) | 0.31 (-0.33, 0.95) | -0.37 (-0.63, -0.11) | -0.14 (-0.51, 0.24) |
| Wealth Quintile 4 | 0.23 (-0.06, 0.40) | 0.26 (-0.45, 0.97) | 0.24 (0.09, 0.40) | 0.50 (-0.21, 1.20) | 0.22 (-0.18, 0.61) | -0.23 (-0.43, -0.02) |
| Wealth Quintile 5 | 0.34 (0.15, 0.53) | -1.88 (-3.15, 0.61) | 0.28 (0.08, 0.47) | 0.20 (-0.75, 1.15) | -0.03 (-0.45, 0.40) | -0.35 (-0.72, 0.01) |
| **Any inpatient visit** | | | | | | |
| Wealth Quintile 1 | 1.95 (1.50, 2.54) | 1.29 (0.61, 2.74) | 1.77 (1.37, 2.28) | 2.32 (0.90, 6.02) | 2.21 (1.62, 3.01) | 2.53 (1.13, 5.68) |
| Wealth Quintile 2 | 2.11 (1.54, 2.91) | 1.33 (0.71, 2.51) | 1.80 (1.43, 2.27) | 1.20 (0.74, 1.93) | 1.42 (1.10, 1.84) | 2.22 (1.47, 3.36) |
| Wealth Quintile 3 | 1.80 (1.44, 2.24) | 1.07 (0.60, 1.90) | 1.43 (1.13, 1.80) | 1.01 (0.49, 2.08) | 1.88 (1.34, 2.63) | 2.41 (1.63, 3.57) |
| Wealth Quintile 4 | 2.06 (1.45, 2.93) | 1.23 (0.70, 2.16) | 1.62 (1.28, 2.04) | 2.13 (1.51, 3.01) | 1.50 (1.21, 1.86) | 1.77 (1.15, 2.73) |
| Wealth Quintile 5 | 1.93 (1.39, 2.69) | 1.52 (0.99, 2.33) | 1.44 (1.19, 1.75) | 2.02 (0.91, 4.48) | 1.35 (0.95, 1.92) | 2.13 (1.42, 3.20) |
| **Hospitalisation days** | | | | | | |
| Wealth Quintile 1 | 0.50 (0.30, 0.69) | 0.27 (-0.34, 0.88) | 0.57 (0.40, 0.75) | 1.57 (-0.24, 3.39) | 0.60 (0.44, 0.76) | 0.55 (-0.04, 1.14) |
| Wealth Quintile 2 | 0.64 (0.43, 0.85) | -0.17 (-0.90, 0.55) | 0.55 (0.35, 0.76) | -0.02 (-0.44, 0.41) | 0.19 (-0.05, 0.42) | 0.46 (0.15, 0.78) |
| Wealth Quintile 3 | 0.66 (0.34, 0.97) | 0.37 (-0.06, 0.80) | 0.35 (0.12, 0.57) | -0.09 (-1.08, 0.90) | 0.34 (0.14, 0.53) | 1.01 (0.55, 1.47) |
| Wealth Quintile 4 | 0.61 (0.35, 0.86) | 0.44 (-0.02, 0.91) | 0.53 (0.33, 0.73) | 1.13 (0.33, 1.93) | 0.27 (0.04, 0.51) | 0.10 (-0.22, 0.41) |
| Wealth Quintile 5 | 0.59 (0.27, 0.91) | 0.50 (0.05, 0.95) | 0.42 (0.21, 0.63) | 1.19 (0.18, 2.20) | 0.31 (0.14, 0.48) | 0.61 (0.38, 0.84) |
| **Inpatient out-of-pocket spending** | | | | | | |
| Wealth Quintile 1 | -0.38 (-1.25, 0.49) | 1.35 (0.06, 2.65) | 0.06 (-0.38, 0.27) | 0.39 (-3.05, 3.84) | 0.60 (-0.08, 1.27) | -0.36 (-1.00, 0.28) |
| Wealth Quintile 2 | -0.24 (-0.49, 0.01) | 1.42 (-0.40, 3.24) | -0.46 (-0.99, 0.08) | 1.99 (0.75, 3.23) | 0.92 (0.10, 1.74) | 0.49 (-0.33, 1.30) |
| Wealth Quintile 3 | 0.28 (-0.08, 0.64) | 0.60 (-1.11, 2.30) | 0.12 (-0.41, 0.65) | 0.45 (-0.10, 0.99) | -0.31 (-0.78, 0.16) | 0.28 (-0.26, 0.82) |
| Wealth Quintile 4 | -0.01 (-0.33, 0.32) | -0.25 (-1.74, 1.24) | -0.01 (-0.25, 0.23) | 0.12 (-1.23, 1.47) | 0.40 (-0.23, 1.04) | 0.37 (-0.13, 0.87) |
| Wealth Quintile 5 | -0.10 (-0.39, 0.18) | -0.68 (-1.65, 0.30) | 0.19 (-0.11, 0.50) | -0.12 (-0.32, 0.08) | 0.83 (0.45, 1.21) | -0.37 (-1.75, 1.01) |

Note: Wealth Quintile 1 is the poorest group, and quintile 5 is the most affluent

**Table E: Percentage of out-of-pocket spending for each type of outpatient service by number of NCDs**

| ***Number of NCDs*** | **Percentage of outpatient out-of-pocket spending (%)** | | | | |
| --- | --- | --- | --- | --- | --- |
|  | **Healthcare provider** | **Medicines** | **Medical Test** | **Transport** | **Others** |
| **China** |  |  |  |  |  |
| 0 | 1.9 | 88.1 | 6.6 | 1.8 | 1.6 |
| 1 | 2.2 | 90.8 | 5.5 | 1.0 | 0.5 |
| 2+ | 6.2 | 83.5 | 8.3 | 1.0 | 1.0 |
| **Ghana** |  |  |  |  |  |
| 0 | 9.3 | 50.5 | 11.0 | 20.1 | 9.2 |
| 1 | 9.1 | 42.8 | 11.8 | 25.7 | 10.6 |
| 2+ | 6.6 | 32.0 | 11.3 | 38.5 | 11.7 |
| **India** |  |  |  |  |  |
| 0 | 13.2 | 73.6 | 3.2 | 7.6 | 2.5 |
| 1 | 15.7 | 66.2 | 5.9 | 9.4 | 2.8 |
| 2+ | 16.0 | 61.0 | 9.5 | 10.9 | 2.5 |
| **Mexico** |  |  |  |  |  |
| 0 | 7.8 | 11.2 | 29.7 | 14.2 | 37.1 |
| 1 | 10.0 | 9.7 | 24.6 | 21.8 | 33.9 |
| 2+ | 13.1 | 15.4 | 23.0 | 18.8 | 29.8 |
| **Russia** |  |  |  |  |  |
| 0 | 34.1 | 42.3 | 4.7 | 18.7 | 0.2 |
| 1 | 26.9 | 60.0 | 1.6 | 9.9 | 1.6 |
| 2+ | 4.2 | 83.5 | 3.4 | 8.7 | 0.1 |
| **South Africa** |  |  |  |  |  |
| 0 | 69.0 | 5.6 | 5.2 | 20.0 | 0.1 |
| 1 | 60.6 | 16.7 | 2.7 | 20.0 | 0.1 |
| 2+ | 65.9 | 13.7 | 0.3 | 20.1 | 0.0 |

**Table F: Percentage of out-of-pocket spending for each type of inpatient service by number of NCDs**

| ***Number of NCDs*** | **Percentage of inpatient out-of-pocket spending (%)** | | | |  |
| --- | --- | --- | --- | --- | --- |
|  | **Health care Provider** | **Medicines** | **Medical Test** | **Transport** | **Others** |
| **China** |  |  |  |  |  |
| 0 | 12.5 | 49.7 | 25.2 | 1.2 | 11.4 |
| 1 | 6.8 | 60.1 | 23.8 | 1.1 | 8.2 |
| 2+ | 11.2 | 60.6 | 17.6 | 2.0 | 8.6 |
| **Ghana** |  |  |  |  |  |
| 0 | 30.4 | 20.6 | 13.4 | 23.5 | 12.1 |
| 1 | 12.0 | 35.5 | 12.0 | 21.1 | 19.4 |
| 2+ | 18.4 | 24.6 | 8.6 | 37.2 | 11.2 |
| **India** |  |  |  |  |  |
| 0 | 12.1 | 55.9 | 9.2 | 11.7 | 11.1 |
| 1 | 10.6 | 52.4 | 12.0 | 12.8 | 12.3 |
| 2+ | 14.5 | 49.2 | 11.8 | 11.0 | 13.6 |
| **Mexico** |  |  |  |  |  |
| 0 | 9.9 | 5.2 | 5.3 | 12.1 | 67.4 |
| 1 | 6.1 | 4.5 | 10.4 | 21.1 | 58.0 |
| 2+ | 14.4 | 17.9 | 22.3 | 17.5 | 27.9 |
| **Russia** |  |  |  |  |  |
| 0 | 5.6 | 23.1 | 1.4 | 9.6 | 60.3 |
| 1 | 1.4 | 14.4 | 22.9 | 31.7 | 29.5 |
| 2+ | 3.2 | 55.8 | 15.5 | 2.9 | 22.6 |
| **South Africa** |  |  |  |  |  |
| 0 | 87.8 | 2.2 | 4.2 | 5.6 | 0.2 |
| 1 | 88.0 | 0.8 | 7.1 | 4.0 | 0.0 |
| 2+ | 73.2 | 1.1 | 1.8 | 12.4 | 11.5 |
